# The Jaga Diri digital intervention improved knowledge and adherence to weekly iron-folic acid supplementation among adolescent girls in Maluku Province, Indonesia

**DOI:** 10.3389/fdgth.2025.1729623

**Published:** 2026-01-22

**Authors:** Lershito Antonio Pasamba, Christiana Rialine Titaley, Sean Samuel Istia, Ritha Tahitu, Elpira Asmin, Farah Christina Noya, Mega Clarita Laurence, Yudhie Djuhastidar Tando, Maxwell Landri Vers Malakauseya, Liyani Sartika Sara

**Affiliations:** 1 Faculty of Medicine, Universitas Pattimura, Ambon, Indonesia; 2 Department of Public Health, Faculty of Medicine, Universitas Pattimura, Ambon, Indonesia; 3 Department of Information and Technology, Faculty of Medicine, Universitas Pattimura, Ambon, Indonesia; 4 Department of Medical Education, Faculty of Medicine, Universitas Pattimura, Ambon, Indonesia

**Keywords:** anaemia, digital health, female students, iron folic acid tablets, mobile health

## Abstract

**Introduction:**

Adherence to weekly iron/folic acid (IFA) supplementation, a vital intervention to combat anaemia among adolescent girls, remains a global challenge, including in Maluku Province, Indonesia. This study assessed the effect of “Jaga Diri” application, in enhancing knowledge and adherence to IFA supplementation among adolescent girls from Salahutu Sub-District of Maluku Province, Indonesia.

**Methods:**

In mid-2024, a quasi-experimental study was conducted among 82 adolescent girls from two senior high schools in Salahutu Sub-District, Maluku Province, Indonesia. The intervention group used the “*Jaga Diri*” Android-based application for four weeks, which delivered weekly reminders and brief educational messages on anaemia and iron–folic acid (IFA) supplementation, while the control group received routine school-based services. Knowledge was measured using a validated 15-item questionnaire. Adherence was defined as consumption of ≥75% of the provided weekly IFA tablets over the previous four weeks, assessed by self-report, and supported by haemoglobin measurement. Group differences were analyzed using non-parametric and chi-square tests, and multivariable binary logistic regression was used to assess factors associated with high knowledge and adherence.

**Results:**

After four weeks of using the “Jaga Diri” application, adolescent girls from the intervention school showed a significantly higher level of knowledge about anaemia (*p* = 0.011) and adherence to weekly IFA supplementation (*p* < 0.001) than those from the control school. The improved adherence was shown by the reduction of anaemia prevalence in the intervention school, from 35% to 17.5%. In the control school, the prevalence increased from 19% to 28.6%.

**Conclusions:**

The “Jaga Diri” application effectively improves knowledge about anaemia and adherence to IFA supplementation among adolescent girls. Further investigation with larger and more varied groups are required to confirm its effectiveness before it can be widely implemented in larger areas of Maluku and Indonesia.

## Introduction

Anaemia is a blood disorder characterized by a lack of red blood cells and haemoglobin (Hb) in the body (1). The Hb plays a vital role in binding oxygen and transporting it throughout the body via the bloodstream. In anaemic conditions, reduced levels of Hb impair the blood's ability to carry oxygen, disrupting the body's functions and overall performance (2).

According to the World Health Organization, approximately one-third of women of reproductive age worldwide—equivalent to around 539 million women aged 15–49 years; experience anaemia (2). The prevalence of anaemia varies widely across countries. In Indonesia, data from the 2023 Indonesian Health Survey (3) reported that anaemia affected 16.3% of adolescent girls aged 5–14 years and 15.5% of those aged 15–24 years. This indicates an improvement compared to the 2018 survey, in which affected 26.8% of adolescent girls aged 5–14 years and 30.2% of those aged 15–24 (4, 5).

The Government of Indonesia has undertaken substantial initiatives to reduce anaemia prevalence, particularly among adolescent girls, including the introduction of weekly IFA supplementation in 2014 (6). However, studies indicated that the distribution and uptake of these supplements among adolescent girls remain significantly below the targeted levels (7, 8). These efforts are further hindered by low motivation and poor adherence among adolescent girls, undermining the effectiveness of anaemia prevention strategies (9). There were several factors reported to be associated with adolescent girls' adherence to IFA supplementation, such as parents' level of education (9, 10), cues to action (9), motivations to consume IFA tablets, as well social supports (10) including supports from school (9, 11).

One potential approach to improve adherence to IFA supplementation is digital interventions (12). In the current era, digital interventions are gaining popularity due to their effectiveness and ability to be tailored to individual needs (13, 14). Several studies have reported the positive impact of digital including mobile interventions on adherence to IFA supplementation among young women in Indonesia (15–17). Meta-analytic evidence also suggested the effectiveness of digital interventions of (e.g., mHealth and messaging-based approaches) in improving adherence to IFA supplementation and haemoglobin levels, with potential implications for reducing anaemia risk (18, 19). These interventions may work through repeated reminders and accessible health education that strengthen knowledge and sustain adherence behaviours.

Maluku, one of Indonesia's largest archipelagic regions, faces unique challenges in healthcare delivery due to its geographic dispersion and limited infrastructure. A previous study conducted in several senior high schools in Ambon City, the capital of Maluku, revealed low adherence to weekly IFA supplementation, with only 15% of students complying, while the prevalence of anaemia was alarmingly high at nearly 50% (20–23). Digital interventions could, therefore, offer a practical solution by bridging these gaps through mobile technology. Such tools can deliver consistent health messages and reminders directly to users, ensuring that even those in remote islands could access critical health information and support. Moreover, leveraging digital platforms could address the shortage of health workers in reaching far and underserved areas as health services could be provided remotely, reducing the need for physical presence and ensuring support for communities in isolated areas.

In early 2024, a research team from the Faculty of Medicine at Universitas Pattimura developed the “Jaga Diri”, an android-based application*,* serving as a reminder to consume IFA tablets. Following four weeks of implementation, an evaluation was conducted to assess its effectiveness. This study was designed as a pilot, school-based quasi-experimental intervention to assess the feasibility and preliminary effectiveness of a digital health application in a real-world setting. Two schools were purposively selected based on operational feasibility, comparable characteristics, and collaboration with local health and education authorities. While this non-randomized approach allowed implementation under routine conditions, it may introduce selection bias and limit generalisability, which should be considered when interpreting the findings. The objective of this analysis was to determine the impact of the “Jaga Diri” application on improving knowledge about anaemia and enhancing adherence to IFA supplementation among adolescent girls in Salahutu Sub-district, Maluku Province, Indonesia.

## Materials and methods

### Research design and study site

This research was carried out from May to June 2024, in two senior high schools, i.e., SMA Negeri 5 Central Maluku (intervention) and SMA Negeri 47 Central Maluku (control), located in Salahutu Sub-District, Maluku Province. We used an analytical design with a quasi-experimental approach. The control school did not receive the “Jaga Diri” application intervention, while the intervention school was provided with the application as part of the intervention. This study was conducted in two schools in Salahutu Sub-District, Maluku Province: SMA Negeri 5 Central Maluku served as the intervention school, while SMA Negeri 47 Central Maluku served as the control school.

### Population and sampling

The senior high schools were selected using purposive sampling based on the following criteria: (1) located in Salahutu Sub-District of Central Maluku District; (2) government-owned school; (3) had a minimum enrolment of 100 female students (grades X to XII); (4) received Weekly IFA from local community health centres; (5) sufficient distance from each other to minimize the risk of contamination; and (6) willingness to participate in the study. Of the five government-owned senior high schools in Salahutu Sub-District, two schools (SMA Negeri 5 Central Maluku and SMA Negeri 47 Central Maluku) were selected for this study—one as the intervention and the other as the control school (24).

The inclusion criteria for study subjects were: (1) female students from Grades X and XI, and (2) willing to participate in the study. For the intervention school, subjects should have a communication device (smartphone, tablet, or laptop) with regular internet access and were willing to install the application.

**Figure 1:**
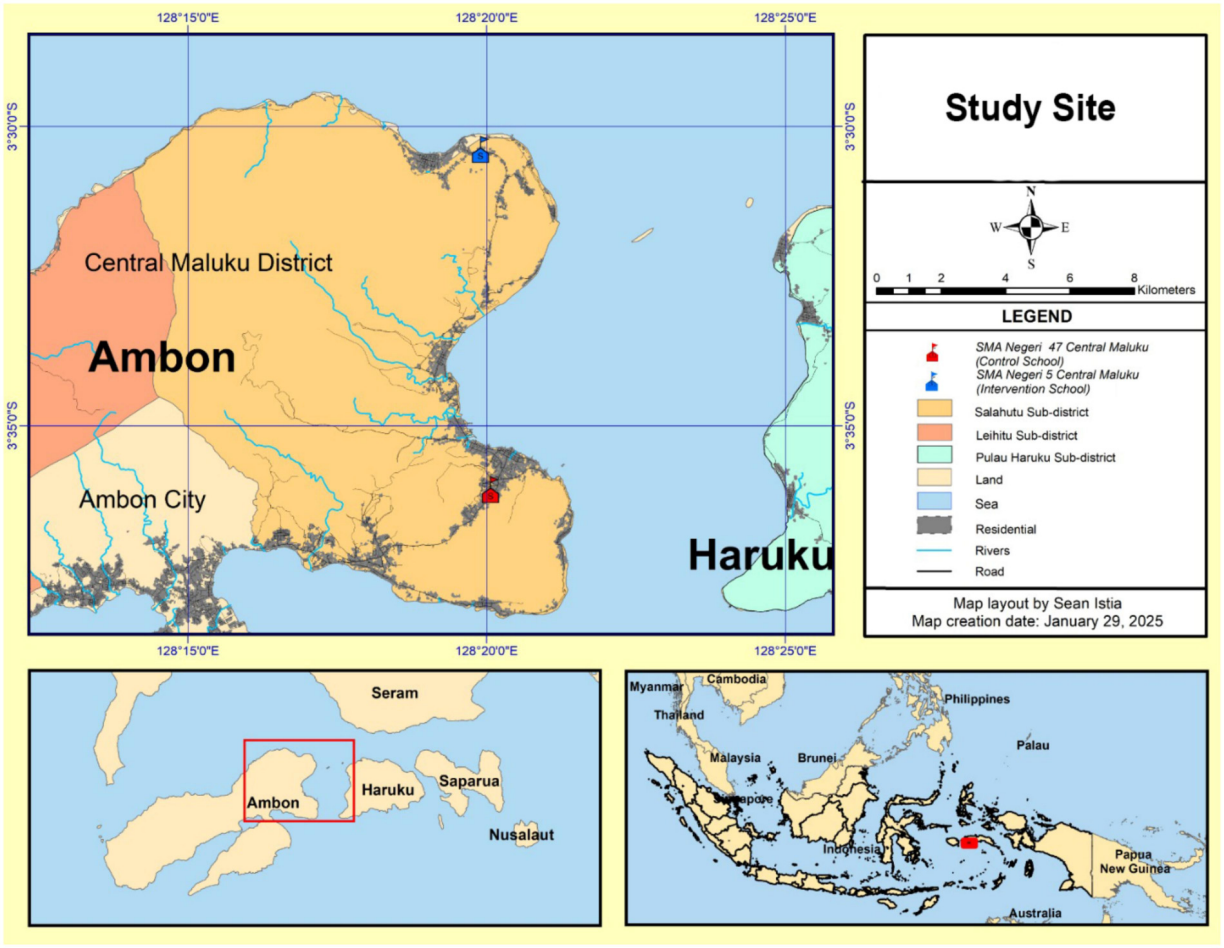
Map of study site.

This study was conducted as a pilot quasi-experimental study, and the sample size was determined by the available number of eligible students in the participating schools. In total, there were 40 adolescent girls from SMAN 5 Central Maluku (intervention school) and 42 from SMAN 47 Central Maluku (control school) included (Figure 1).

**Figure 2:**
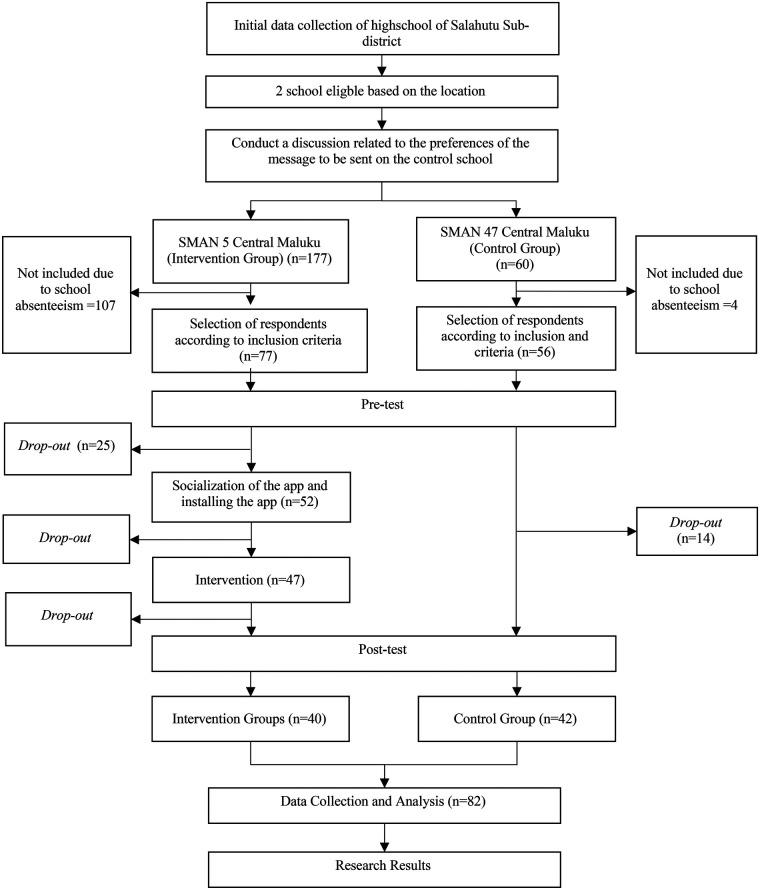
Research flow chart.

No formal exclusion criteria were defined; non-participation and loss to follow-up occurred due to operational reasons such as school absenteeism and technical constraints. In the control group, the decline in participants in the study was due to their absence during data collection, both at the beginning and the end of the study. Meanwhile, in the intervention group, the decrease in participants was attributed to various reasons corresponding to different stages of the study. For instance, during the pre-test phase, 107 participants were excluded due to school absenteeism, as a local government event related to scholarships required their attendance on very short notice, making it impossible to reschedule the data collection. Furthermore, 25 participants dropped out after the pre-test due to technical issues with their devices, which prevented the application from being installed before its explanation could be provided (Figure 2).

### Intervention

In this study, adolescent girls from the intervention school were required to install and use the “Jaga Diri” application and receive routine services provided by the local health centre. The “Jaga Diri” application, developed in 2024 by the Faculty of Medicine at Universitas Pattimura, is a reminder tool for taking IFA supplementation. It incorporates a reminder alarm commonly used on smartphones, enhanced with features that deliver health messages to educate users about anaemia and the importance of IFA supplementation. Currently, this application is exclusively available on the Android platform. Unlike other anaemia intervention apps, such as Aneminfo (16) and TeenFit (17) focusing mainly on providing educational content to encourage compliance indirectly, “Jaga Diri” also integrates a direct reminder feature to prompt users to take their IFA tablets regularly. This combination of direct reminders and educational support sets it apart from applications that rely solely on improving knowledge to enhance adherence among adolescent girls (a detailed explanation of the “Jaga Diri” application is provided in Supplementary File 1). Students in the control school received only the routine services provided by the local health centre, in accordance with national guidelines. These services consisted of brief health education sessions delivered by health workers or teachers, together with general monitoring of tablet distribution records. No digital reminders, structured follow-up, or individualized adherence monitoring were included as part of the routine services. To ensure a consistent supply, IFA tablets were distributed by the research team to all schools involved in the study (both control and intervention).

### Evaluation method

There were two cross-sectional surveys carried out: one before the intervention (as baseline) and another after the intervention (as endline). Both surveys used structured questionnaires to assess adherence to IFA supplementation and knowledge about anaemia and IFA supplementation. The adherence questionnaire comprised four items addressing IFA consumption over the previous four weeks. Using a four-week period for this evaluation aligns with studies by Kang and Park (25), Riegel (26), and Goldstain (27). Some studies even used shorter evaluation periods, such as three weeks or as brief as 2 h (28).

To assess the level of knowledge, a questionnaire consisting of 15 questions was administered, focusing on general information about anaemia and the significance of IFA supplementation. The questionnaire used in this study was adopted from another study conducted in Indonesia that has also been tested for validity and reliability (29). The blood Hb test was performed using the HemoCue HB 301 device to determine the anaemia status of adolescent girls. In this study, the involvement of teachers was limited to avoid potential bias in data collection and to ensure the independence and objectivity of responses. The questionnaire was administered through face-to-face interviews by trained enumerators at both baseline and endline to ensure consistent understanding of the questions.

### Outcome variables

The outcome variables of this study were: (1) the level of knowledge about anaemia and (2) adherence to IFA consumption among adolescent girls. Adherence referred to the practice of consuming at least 75% of the IFA tablets provided, i.e., equivalent to three out of four tablets. Blood Hb levels were also measured to validate adherence to IFA supplementation.

We assessed adolescent girls' knowledge and awareness about anaemia and weekly IFA supplementation using 15 questions that covered different topics including the signs and symptoms of anaemia, the benefits of IFA supplementation, recommended consumption practices, and its possible side effects. Each correct answer was given a score of 1, while incorrect answers received a score of 0. The level of knowledge was classified as high or low according to the median score. Participants scoring above the median categorized as having high knowledge and those scoring below categorized as having low knowledge.

### Data analysis

Descriptive analyses were conducted to summarise baseline characteristics using frequencies and percentages. The normality of continuous variables, including knowledge scores and haemoglobin levels, was assessed using the Kolmogorov–Smirnov test. As these variables were not normally distributed, between-group comparisons at baseline and endline were performed using Mann–Whitney *U* tests. Differences in categorical outcomes, including anaemia status and adherence to weekly IFA supplementation, were analysed using chi-square tests, after confirming that the expected cell counts met the assumptions required for this analysis.

Multivariable binary logistic regression analyses were conducted to assess factors independently associated with (1) high knowledge of anaemia and IFA supplementation and (2) adherence to weekly IFA supplementation at endline. Models were adjusted for a limited number of pre-specified covariates, including age and relevant baseline characteristics, to control for potential confounding while avoiding overfitting. Adjusted odds ratios (aORs) with 95% confidence intervals were reported. All analyses were performed using IBM SPSS Statistics for Windows, version 23.

### Ethics approval

Ethical approval was granted by the Ethics Commission of the Faculty of Medicine, Universitas Pattimura, under approval letter number 014/FK-KOM.ETIK/VIII/2024. Prior to data collection, the objectives of the study were clearly explained to all participants, and written informed consent was obtained. Their voluntary participation was emphasized, with the option to decline or withdraw at any time. The confidentiality and anonymity of all participant information were strictly maintained.

## Results

The distribution of 82 respondents (40 in intervention schools and 42 in control schools) by age and anaemia status at the beginning of the study is presented in Table 1. The proportion of anaemia in adolescent girls was higher in the intervention school (35%) than control school (19%).

**Table 1:** Frequency distribution of subjects by age and of anaemia status in control and intervention school.

Variable	Intervention (*n* = 40)		Control (*n* = 42)	
	*n*	%	*n*	%
Age (years)				
15	17	42.5	5	11.9
16	17	42.5	21	50.0
17	4	10.0	10	23.8
18	2	5.0	6	14.3
Paternal occupation				
Formal	6	15.0	2	4.7
Informal	28	70.0	34	81.0
Don't know/not working	6	15.0	6	14.3
Paternal highest educational attainment				
Completed primary school	5	12.5	6	14.3
Completed junior high school	4	10.0	2	4.7
Completed senior high school	16	40.0	23	54.8
Academy/University	4	10.0	0	0
Don't know	11	27.5	11	26.2
Maternal occupation				
Formal	5	12.5	2	4.7
Informal	32	80.0	37	88.1
Don't know	3	7.5	3	7.2
Maternal highest educational attainment				
Completed primary school	4	10.0	7	16.7
Completed junior high school	3	7.5	4	9.5
Completed senior high school	18	45.0	18	42.9
Academy/University	5	12.5	4	9.5
Don't know	10	25.0	8	19.0
Menarche status				
Not yet	1	2.5	1	2.4
Yes	39	97.5	41	97.6
Ever heard of anaemia				
No	14	35.0	17	40.5
Yes	26	65.0	25	59.5
Status of anaemia				
Anaemia (<12 g/dL)	14	35.0	8	19.0
Non-Anaemia (≥12 g/dL)	26	65.0	34	81.0
Body Mass Index				
Underweight (<18.5 kg/m^2^)	19	47.5	23	4.8
Normal (18.5–<25 kg/m^2^)	20	50.0	17	40.5
Overweight/obese (≥25 kg/m^2^)	1	2.5	2	4.8

**Figure 3:**
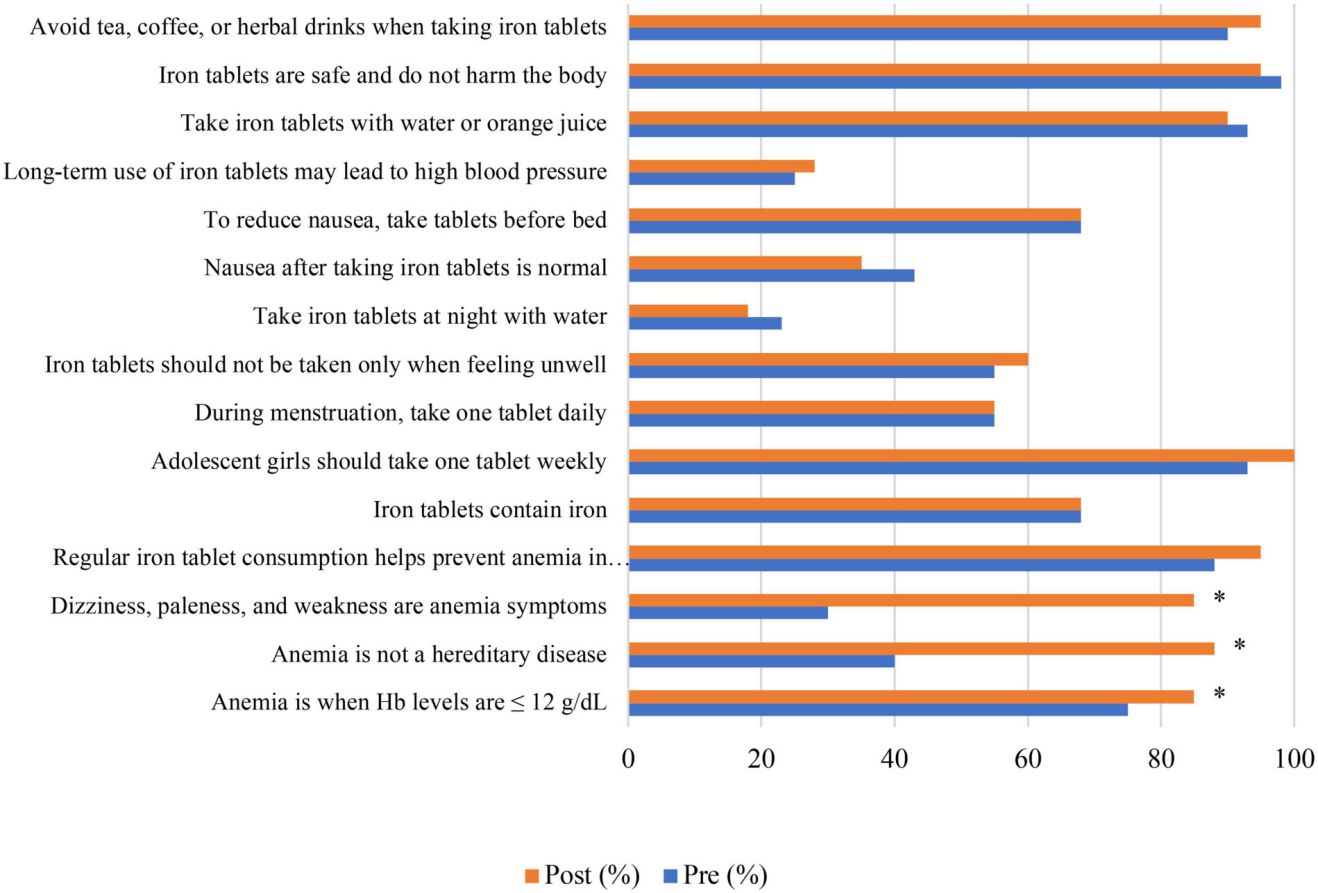
Distribution of correct answers for each knowledge question Among female students in the intervention school. *McNemar value <0.05.

**Figure 4:**
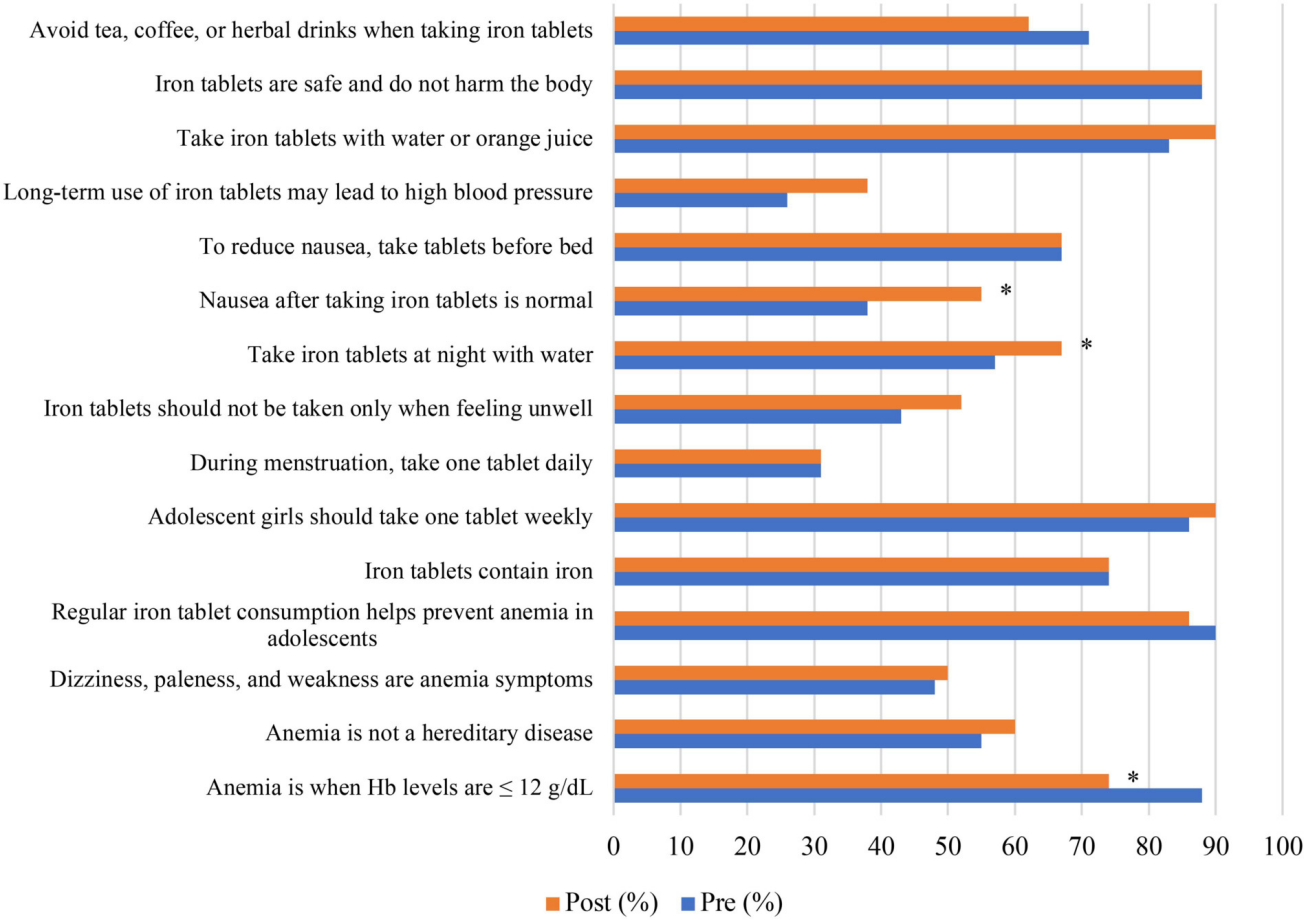
Distribution of correct answers for each knowledge question Among female students in the control school. *McNemar value <0.05.

Table 2 presents differences in knowledge levels regarding anaemia and iron–folic acid (IFA) supplementation among adolescent girls at baseline and endline in both the intervention and control schools. In the intervention school, the proportion of adolescents with a high level of knowledge increased significantly from 47.5% at baseline to 85.0% at endline (*p* < 0.001). In contrast, no significant change in knowledge level was observed in the control school between baseline and endline (*p* = 0.510). At baseline, mean knowledge scores were comparable between the intervention and control groups (9.40 vs. 9.45). By endline, however, the intervention group demonstrated a higher mean knowledge score than the control group (10.63 vs. 9.83), indicating greater improvement following the intervention. The distribution of correct responses for each knowledge item in the intervention and control schools at baseline and endline is shown in Figures 3, 4, respectively.

**Table 2:** The difference between the level of knowledge about anaemia and IFA supplementation between subjects in control and intervention school at baseline and endline.

Group	Level of knowledge^a^ about anaemia				*p-value*
	Low		High		
	*n* ^b^	%	*n* ^b^	%	
Intervention					
Baseline	21	52.5	19	47.5	<0.001
Endline	6	15.0	34	85.0	
Control					
Baseline	20	47.6	22	52.4	0.510
Endline	17	40.5	25	59.5	

a Knowledge was categorised as *low* or *high* based on the median score of the study population. Scores below the median classified as low knowledge and scores equal to or above the median classified as high knowledge.

b The number of participants included in the analysis.

Table 3 presents the differences in adolescent girls' adherence to weekly IFA supplementation (defined as consuming at least 75% of the tablets provided) before and after the implementation of the “Jaga Diri*”* application. In the control schools, there was no significant difference in adherence between baseline (4.8%) and endline (7.1%) (*p* = 1.000). In contrast, the intervention schools showed a significant increase in adherence, with the proportion of adolescent girls adhering to weekly IFA supplementation rising from 5.0% at baseline to 80.0% at endline (*p* < 0.001).

**Table 3:** The difference between adherence to weekly IFA supplementation between subjects in control and intervention school at baseline and endline.

Group	Adherence^a^		Non-adherence		*p*-Value
	*n*	%	*n*	%	
Intervention					
Baseline	2	5.0	38	95.0	<0.001
Endline	32	80.0	8	20.0	
Control					
Baseline	2	4.8	40	95.2	1.000
Endline	3	7.1	39	92.9	

a Adherence referred to the practice of consuming at least 75% of the IFA tablets provided (three out of four tablets).

These findings were also confirmed when we compared the levels of knowledge and adherence between the intervention and control groups at endline (Table 4). A statistically significant difference was observed in the proportion of adolescent girls with a high level of knowledge and awareness about anaemia and IFA supplementation (*p* = 0.006), as well as adherence to weekly IFA supplementation (*p* < 0.001).

**Table 4:** Comparations the levels of knowledge and adherence between the intervention and control groups at endline.

Variable	Intervention		Control		*p*-value
	*n* ^a^	%	*n* ^a^	%	
High level of knowledge	25	59.5	33	86.8	0.006
Adherence	30	78.9	3	7.1	<0.001

a The number of participants included in the analysis.

Based on the multivariable binary logistic regression analysis (Table 5), adolescents in the intervention group had significantly higher odds of having high knowledge compared with those in the control group [adjusted odds ratio (aOR) = 4.94, *p* = 0.011], after adjustment for age and baseline knowledge. Baseline knowledge was also a significant predictor of endline knowledge (aOR = 5.24, *p* = 0.005), whereas age was not independently associated with knowledge. The model showed acceptable fit (Nagelkerke *R*^2^ = 0.25).

**Table 5:** Results of multivariable binary logistic regression to assess factors associated with high knowledge and adherence.

Variable	High level of knowledge^a^				Adherence^b^			
	aOR	95% CI		*p*-value	aOR	95% CI		*p*-value
Group								
Control	1.00				1.00			
Intervention	4.94	1.44	16.95	*0*.*011*	44.53	10.49	189.07	<0.001
Age (in years)	1.00	0.53	1.89	*<0*.*001*	0.59	0.25	1.39	0.229
Knowledge level at baseline								
Low level								
High level	5.24	1.66	16.52	*0*.*005*				
Adherence at baseline								
Non-adherence					1.00			
Adherence					0.49	0.02	10.39	0.649

a Knowledge was categorised as *low* or *high* based on the median score of the study population. Scores below the median classified as low knowledge and scores equal to or above the median classified as high knowledge.

b Adherence referred to the practice of consuming at least 75% of the IFA tablets provided (three out of four tablets).

Furthermore, adolescents in the intervention group also had significantly higher odds of adherence compared with those in the control group (aOR = 44.53, *p* < 0.001), after adjusting for age and baseline adherence (Table 5). Neither age nor baseline adherence was independently associated with adherence in the adjusted model. The model demonstrated good fit (Nagelkerke *R*^2^ = 0.64). To confirm adherence, we also evaluated the difference in the proportion of anaemia between female students in intervention and control schools. Our study found that in the intervention school, the proportion of anaemia reduced from 35% to 17.5%, while in the control, the proportion increased from 19% to 28.6%.

**Figure 5:**
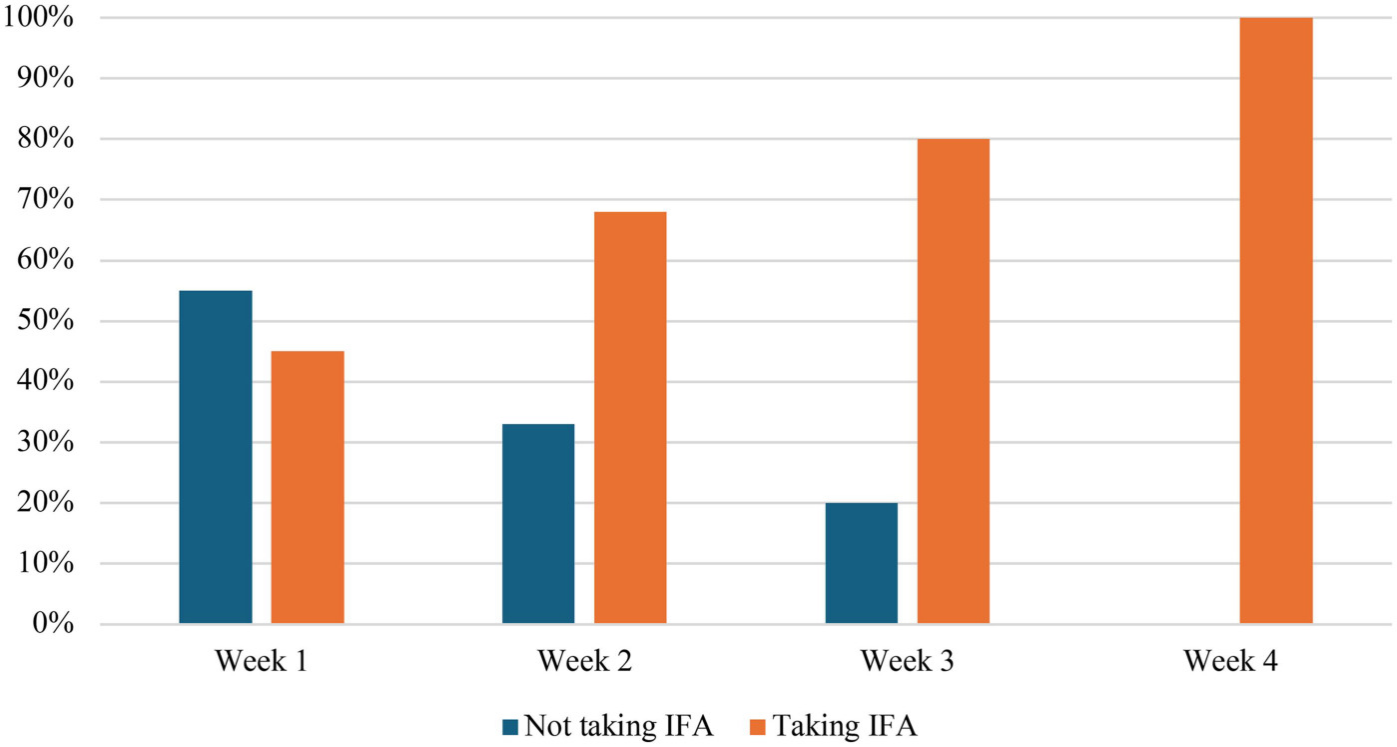
Percentage of taking IFA each week after intervention (intervention school).

This study also examined changes in the weekly consumption rate of IFA supplementation after the intervention, as illustrated in Figure 3. Initially, 45% of adolescent girls in the intervention school consumed the tablets, while 55% did not. Over time, adherence improved progressively with continued use of the “Jaga Diri*”* application. Adherence rates increased from 68% in the second week to 80% in the third week, reaching 100% by the final week of the study (Figure 5).

## Discussion

The study found that the “Jaga Diri” application significantly improved knowledge about anaemia and adherence to weekly IFA supplementation among adolescent girls. Adolescent girls in the intervention school demonstrated improved knowledge about anaemia and its prevention compared to those from the control school. The intervention also led to a steady increase in the consumption of IFA supplementation over the study period, with adherence rates being substantially higher in the intervention school. This study emphasizes the promise of mobile health interventions, such as the “Jaga Diri” application, in improving knowledge and adherence to IFA supplementation among adolescent girls. The results highlight the need to integrate technology-based approaches into public health initiatives to more effectively prevent and control anaemia.

The “Jaga Diri” application was aimed to improve adherence to IFA supplementation and enhance knowledge about anaemia prevention among adolescent girls. The existing applications for IFA interventions generally focused on a single objective: improving knowledge (indirectly enhancing adherence) or serving as a reminder alarm to encourage compliance. In contrast, the “Jaga Diri” application integrates both functions—it not only acts as a reminder or alarm for taking iron-folic acid tablets but also serves as a platform to enhance users' knowledge. Another advantage of this application is its flexible knowledge feature, which could be accessed via a website with customizable content. Unlike most IFA educational applications, which are pre-packaged and, although operable offline, tend to offer static and monotonous content, the “Jaga Diri” application provides a dynamic and adaptable learning experience.

We found that the “Jaga Diri” application has a positive influence in improving knowledge in adolescent girls about anaemia. This aligns with previous research (16, 17), which showed increased knowledge about anaemia due to using mobile interventions. The health messages integrated into the application improved adolescent girls' knowledge about anaemia by providing consistent, targeted, and easily digestible information. This consistent exposure helps users internalize important details over time without feeling overwhelmed. Simple messages tailored to the user's needs could address common questions, misconceptions, and the benefits of adherence in a straightforward manner, making complex topics more approachable (30).

The findings of this study highlight the effectiveness of the “Jaga Diri” application in improving adherence to IFA supplementation among adolescent girls. The application's reminder features ensure consistent engagement by providing timely prompts that help users establish a routine for taking supplements. These reminders reinforce the habit of adherence and deliver concise, targeted health messages to enhance understanding of anaemia prevention. Adolescents often face challenges like busy or unstructured schedules, and the reminders help integrate IFA supplementation into their daily activities (31). Furthermore, the reminders are customizable, allowing users to set specific times and frequencies, making the intervention more user-centred and adaptable to individual needs. When paired with motivational and educational messages, the reminders create a supportive system that fosters accountability, emphasizes the importance of adherence, and offers a sustainable and practical solution to overcome barriers such as forgetfulness and lack of routine (12, 15). This combination makes the “Jaga Diri” application a useful tool for enhancing compliance among adolescent girls.

The findings of this study should be interpreted with caution. The quasi-experimental design, inclusion of only one school per study arm, and absence of randomization limit causal inference and raise the possibility of unmeasured confounding. In addition, adherence to weekly IFA supplementation was primarily assessed through self-report, which may be subject to recall and social desirability bias, despite support from haemoglobin measurement. These methodological considerations may affect the internal validity and generalisability of the findings. In line with the WHO guidance, haemoglobin measurement was included as a supportive indicator rather than a definitive confirmation of adherence, as short-term changes in Hb are insufficient to reliably reflect supplement intake or sustained physiological response. Furthermore, the observed improvement in haemoglobin levels over the relatively short intervention period should be interpreted cautiously, as it may partly reflect biological variability or measurement variation rather than sustained physiological improvement.

Nevertheless, this study also found that, despite the significant impact of the “Jaga Diri” application in improving adherence, some adolescents in the intervention school (20%) still did not comply with consuming weekly IFA supplementation. This non-compliance aligns with findings from Ningtyias et al. (32), that identified factors such as laziness, boredom, dislike of the tablet's taste, or side effects like nausea as common reasons for not taking weekly IFA supplements. Further research is needed to explore the underlying reasons for non-compliance among students and develop more targeted strategies for improving adherence.

The “Jaga Diri” application significantly improves IFA adherence in archipelagic regions like Maluku. These regions often face logistical challenges, such as limited healthcare infrastructure, geographic isolation, and inconsistent access to health services, which can hinder the distribution and monitoring of IFA supplementation programs. Mobile health applications overcome these barriers by providing a scalable and accessible platform that reaches users directly, regardless of location. Features such as reminders and educational messages ensure consistent engagement and support, even in areas with limited physical access to healthcare providers (33). Additionally, the offline functionality of the “Jaga Diri” application is particularly advantageous in regions with intermittent internet connectivity, ensuring uninterrupted use. Digital interventions can play a crucial role in addressing anaemia and improving overall health outcomes in geographically dispersed communities by empowering adolescents with the tools and knowledge to manage their health.

This study demonstrates several key strengths. It tackles a pressing public health issue, i.e., anaemia among adolescent girls, using the innovative “Jaga Diri” application, which integrates reminder features and educational messages, aligning with the increasing relevance of mobile technology in health interventions. The quasi-experimental design, with baseline and endline evaluations, ensures a thorough assessment of the application's impact on knowledge and adherence to IFA supplementation. The study enhances its methodological rigour by incorporating objective measures such as Hb levels alongside self-reported data.

### Strengths and limitations

Sample attrition might have impacted the study's statistical power to detect differences. To reduce sample loss in future studies, it is advisable to strategically plan data collection schedule, such as at the start of the school term or academic year. The real-world application of the intervention in a school setting underscores its practical feasibility while focusing on Maluku Province, a geographically challenging and underrepresented area. It adds value by providing insights applicable to other resource-limited regions. Despite these strengths, several limitations should be acknowledged. The study was conducted in only two schools, with one school per study arm, which increases the possibility of unmeasured confounding, and restricts the generalisability of the findings. The requirement for smartphone access in the intervention group might have introduced selection bias, as those without compatible devices were unable to participate. In addition, substantial participant attrition due to school absenteeism and technical constraints may have further affected representativeness. The short follow-up period also limited the ability to assess sustained adherence and longer-term behavioural change. Moreover, the “Jaga Diri” application is currently compatible only with Android devices, restricting accessibility for users of other operating systems. Although offline functionality is beneficial in low-connectivity settings, it limits opportunities for real-time monitoring and interaction between users and researchers. Thereby, future research should involve larger and more diverse populations and longer follow-up periods.

## Conclusions

This study demonstrates the significant impact of the “Jaga Diri” application in improving knowledge about anaemia and adherence to weekly IFA supplementation among adolescent girls. The application effectively combines reminder features with targeted health messages, fostering consistent engagement and enhancing understanding of anaemia prevention. Over time, its use increased adherence rates and a notable reduction in anaemia prevalence in the intervention group. The flexibility of the application, including its offline functionality and customizable content, makes it particularly suitable for geographically dispersed and resource-limited areas like Maluku. Despite its success, some users remained non-compliant, highlighting the need for further research into addressing barriers such as side effects, boredom, and lack of motivation. However, these findings should be interpreted with caution, as the short intervention duration, participant attrition, and lack of randomization inherent to the quasi-experimental design limit causal inference and generalizability. The study underscores the potential of mobile health interventions as scalable and practical tools to address public health challenges, particularly in archipelagic regions. Future developments should focus on expanding the application's compatibility and features while validating its effectiveness in broader and more diverse populations to maximize its impact and sustainability.

However, these findings should be interpreted with caution, as the short intervention duration, participant attrition, and lack of randomization inherent to the quasi-experimental design limit causal inference and generalisability.

## Data Availability

The raw data supporting the conclusions of this article will be made available by the authors, without undue reservation.

## References

[1] Turner J Parsi M Badireddy M. Anemia. In: StatPearls. Treasure Island, FL, United States: StatPearls Publishing (2024). Available online at: http://www.ncbi.nlm.nih.gov/books/NBK499994/

[2] World Health Organization. Weekly Iron and Folic Acid Supplementation as An-Anaemia Prevention Strategy in Women and Adolescents Girls—Lessons Learnt from Implementation of Programmes Among Non-Pregnant Women of Reproductive Age. Switzerland: WHO (2018). (WHO/NMH/NHD/18.8). Available online at: https://iris.who.int/bitstream/handle/10665/274581/WHO-NMH-NHD-18.8-eng.pdf?sequence=1

[3] Ministry of Health Republic Indonesia. Indonesian Health Survey. Jakarta: Kemenkes RI (2024). Available online at: https://www.badankebijakan.kemkes.go.id/hasil-ski-2023/

[4] Ministry of Health Republic of Indonesia. National Basic Health Research Report 2018. Jakarta: BALITBANGKES (2019).

[5] Sekartini R Widjaja NA Manikam NRM Jo J Basrowi RW Dilantika C. Iron-Deficiency Anemia: Indonesia’s striving. Asia Pac J Paediatr Child Health. (2022) 5:3–16. Available online at: https://www.apjpch.com/pdfs/2297hJz080011.pdf

[6] Ministry of Health Republic Indonesia. Google Docs. Guidelines for administering Blood Supplement Tablets for adolescent girls during the COVID-19 pandemic for health workers (Pedoman pemberian Tablet Tambah Darah (TTD) bagi remaja putri pada masa pandemi COVID-19 bagi tenaga kesehatan) (2020). Available online at: https://drive.google.com/file/d/1WgNCQconUnaie9M16qFq_oAiC-i2qSLj/view?usp=sharing&usp=embed_facebook (Accessed November 21, 2024).

[7] Helmyati S Syarifa CA Rizana NA Sitorus NL Pratiwi D. Acceptance of iron supplementation program among adolescent girls in Indonesia: a literature review. Amerta Nutr. (2023) 7(3SP):50–61. 10.20473/amnt.v7i3SP.2023.50-61

[8] Ansari MR Kandarina BI Kusmayanti N Fikrinnisa R The acceptability of weekly iron-folic acid supplementation and its influencing factors among adolescent school girls in Yogyakarta city: a mix-methods study. Malays J Nutr. (2021) 27(1):53–66. 10.31246/mjn-2020-0019

[9] Feriyanti A Deviatin NS Nurmala I Widati S Atmaka DR. Determinant of adherence to iron supplementation in adolescent girl in spesific intervention for stunting prevention: systematic review. Media Gizi Indones. (2022) 17:90–6. 10.20473/mgi.v17i1SP.90-96

[10] Silitonga HTH Salim LA Nurmala I Hargono R Notobroto HB Hartini N The role of social support and interpersonal trust to improve compliance of iron supplementation amongst adolescent girls: a qualitative approach. Niger Postgrad Med J. (2023) 30(1):75–80. 10.4103/npmj.npmj_277_22 36814167

[11] Apriningsih MS Dwiriani CM Kolopaking R. Determinant of highschool girl adolescent’ adherence to consume iron folic acid supplementation in Kota Depok. J Nutr Sci Vitaminol (Tokyo). (2020) 66(Suppl):S369–75. 10.3177/jnsv.66.S369 33612627

[12] Choudhury A Shahsavar Y Sarkar K Choudhury MM Nimbarte AD. Exploring perceptions and needs of mobile health interventions for nutrition, anemia, and preeclampsia among pregnant women in underprivileged Indian communities: a cross-sectional survey. Nutrients. (2023) 15(17):3699. 10.3390/nu15173699 37686731 PMC10490056

[13] Shrivastava TP Goswami S Gupta R Goyal RK. Mobile app interventions to improve medication adherence among type 2 diabetes mellitus patients: a systematic review of clinical trials. J Diabetes Sci Technol. (2023 17(2):458–66. 10.1177/19322968211060060 34861793 PMC10012382

[14] Laksono EB Johan A Erawati M. The utilization of mobile-health intervention in improving treatment compliance behavior in Tuberculosis patients. Nurse Health J Keperawatan. (2022) 11(2):275–86. 10.36720/nhjk.v11i2.339

[15] Pratiwi RS Aritonang I Iskandar S. Effectiveness of the “ARMi” application on adolescent girls’ compliance with taking blood supplement tablets (Efektivitas Aplikasi “ARMi” Terhadap Kepatuhan Minum Tablet Tambah Darah Pada Remaja Putri). Med Respati J Ilm Kesehat. (2023) 18(4):297–306. 10.35842/mr.v18i4.980

[16] Saraswati RS Kartini A Agushybana F. The effect of the aneminfo android application on the knowledge and attitudes of adolescent women related to iron deficiency anemia (Pengaruh Aplikasi Android Aneminfo terhadap Pengetahuan dan Sikap Remaja Putri terkait Anemia Defisiensi Besi). J Promosi Kesehat Indones. (2020) 15(2):65–9. 10.14710/jpki.15.2.65-69

[17] Rohani T Diniarti F Febriawati H. The “TEENFIT” smartphone application in increasing adherence to taking iron supplements in adolescents in bantul Indonesia (Aplikasi Smartphone “TEENFIT” Dalam Meningkatkan Kepatuhan Minum Suplemen Zat Besi Pada Remaja Di Bantul Indonesia). J Kesmas Kesehat Masy Khatulistiwa. (2022) 9(3):156–67. 10.29406/jkmk.v9i3.3253

[18] Shao Y Meng C Liang Y-Z. Digital versus non-digital health interventions to improve iron supplementation in pregnant women: a systematic review and meta-analysis. Front Med. (2024) 11:1–9. 10.3389/fmed.2024.1375622 PMC11173591 38873205

[19] Nuryuliana N. Meta-analysis: effectiveness of mhealth use on compliance with iron and folic acid supplement intake in pregnant women. J Vent. (2024) 3(1):28–41. 10.59680/ventilator.v3i1.1709

[20] Titaley CR Tahittu R Asmin E. The 2022 Anemia Research Report. Ambon: Faculty of Medicine Pattimura University (2022).

[21] Sharma S Smitha MV Balakrishnan D. Telephonic intervention to combat non-adherence to oral iron-folic acid supplementation in pregnancy: a randomized controlled trial. Eur J Obstet Gynecol Reprod Biol X. (2023) 20:100235. 10.1016/j.eurox.2023.100235 37736306 PMC10509657

[22] Laurence MC Titaley CR Tahitu R Asmin E Kailola NE Istia SS The effect of WhatsApp-based reminders on enhancing knowledge and adherence to weekly iron-folic acid supplementation among adolescent girls in Maluku, Indonesia. Front Digit Health. (2025) 7:1–10. 10.3389/fdgth.2025.1542006 PMC11983285 40212898

[23] Engidaw MT Lee P Fekadu G Mondal P Ahmed F. Effect of nutrition education during pregnancy on iron–folic acid supplementation compliance and Anemia in low- and middle-income countries: a systematic review and meta-analysis. Nutr Rev. (2025) 83(7):e1472–87. 10.1093/nutrit/nuae170 39545365 PMC12166189

[24] Ministry of Education, Culture, Research, and Technology of the Republic of Indonesia. References Data. Number of Education Unit Data per Central Maluku Regency (Jumlah Data Satuan Pendidikan (Dikdas) per Kab. Maluku Tengah) (2024). Available online at: https://referensi.data.kemdikbud.go.id/pendidikan/dikdas/210100/2/all/all/all (Accessed January 17, 2025).

[25] Kang H Park H-A. A Mobile app for hypertension management based on clinical practice guidelines: development and deployment. JMIR MHealth UHealth. (2016) 4(1):e4966. 10.2196/mhealth.4966 PMC4756253 26839283

[26] Riegel B Stephens-Shields A Jaskowiak-Barr A Daus M Kimmel SE. A behavioral economics-based telehealth intervention to improve aspirin adherence following hospitalization for acute coronary syndrome. Pharmacoepidemiol Drug Saf. (2020) 29(5):513–7. 10.1002/pds.4988 32237005 PMC7217735

[27] Goldstein CM Gathright EC Dolansky MA Gunstad J Sterns A Redle JD Randomized controlled feasibility trial of two telemedicine medication reminder systems for older adults with heart failure. J Telemed Telecare. (2014) 20(6):293–9. 10.1177/1357633X14541039 24958355 PMC6957063

[28] Grindrod KA Li M Gates A. Evaluating user perceptions of mobile medication management applications with older adults: a usability study. JMIR MHealth UHealth. (2014) 2(1):e3048. 10.2196/mhealth.3048 PMC4114457 25099993

[29] Pertiwi CS. Determinants of compliance behavior of consumption of blood supplement tablets in adolescent women in Bangsalsari District, Jember Regency (Determinan Perilaku Kepatuhan Konsumsi Tablet Tambah Darah pada Remaja Putri di Kecamatan Bangsalsari Kabupaten Jember) (Skripsi). Universitas Jember, Jember (2019). Available online at: https://repository.unej.ac.id/xmlui/handle/123456789/97922

[30] Syakir S. Influence of nutrition education using animation media on knowledge and attitude about anemia of adolescence girls (Pengaruh Intervensi Penyuluhan Gizi Dengan Media Animasi Terhadap Perubahan Pengetahuan Dan Sikap Tentang Anemia Pada Remaja Putri). ARGIPA Arsip Gizi Dan Pangan. (2018) 3(1):18–25. 10.22236/argipa.v3i1.2446

[31] Waluyo D Hidayanty H Seweng A. The effect of nutrition education of Anemia among improvement of knowledge in adolescents SMA Negeri 21 makassar (Pengaruh Pendidikan Gizi Anemia Terhadap Peningkatan Pengetahuan Pada Anak Remaja SMA Negeri 21 Makassar). J Kesehat Masy Marit. (2019) 1(3):301–6. 10.30597/jkmm.v1i3.8821

[32] Ningtyias FW Quraini DF Rohmawati N. Compliance behavior of adolescent girls in Jember, Indonesia (Perilaku Kepatuhan Konsumsi Tablet Tambah Darah Remaja Putri di Jember, Indonesia). J Promkes Indones J Health Promot Health Educ. (2020) 8(2):154–62. 10.20473/jpk.V8.I2.2020.154-162

[33] Sungkar A Bardosono S Irwinda R Manikam NRM Sekartini R Medise BE A life course approach to the prevention of iron deficiency Anemia in Indonesia. Nutrients. (2022) 14(2):1–8. 10.3390/nu14020277 PMC8780595 35057458

