# Supplementary material for: The Jaga Diri digital intervention improved knowledge and adherence to weekly iron-folic acid supplementation among adolescent girls in Maluku Province, Indonesia
Source: Front Digit Health. 2026 Jan 22;7:1729623. doi: 10.3389/fdgth.2025.1729623 (PMC12872849; doi:10.3389/fdgth.2025.1729623)
Supplement: Supplementary file 1 [file Datasheet1.pdf]

Supplementary Material

Supplementary Figures

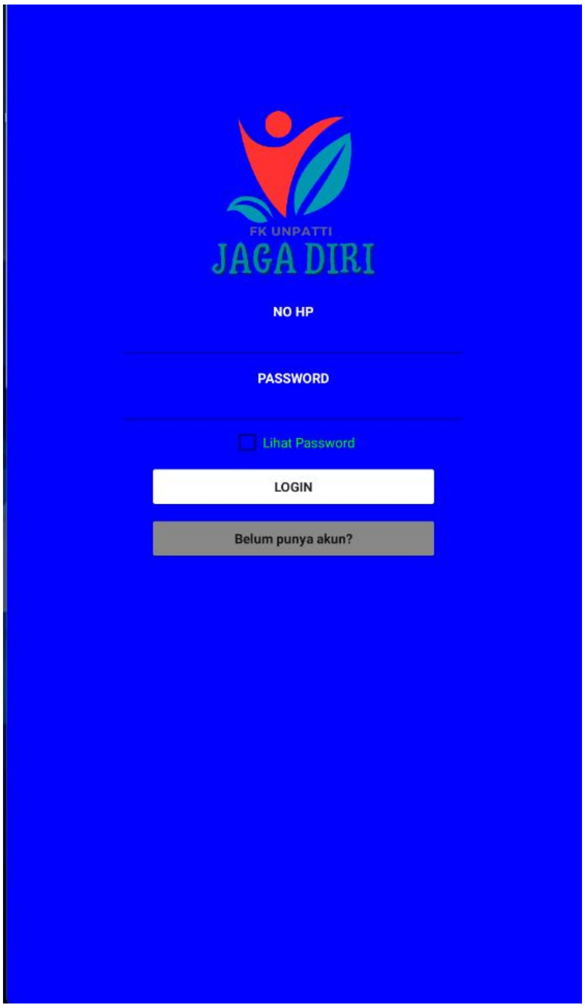

Figure 1. Login Page Display

Figure 1 shows the initial display of the "Jaga Diri" application upon launch, which is the login screen. Users are required to enter their account information, including their cellphone number and password, to access the main menu. If a user does not have a "Jaga Diri" account, they can register by clicking the 'Don't have an account yet?' button.

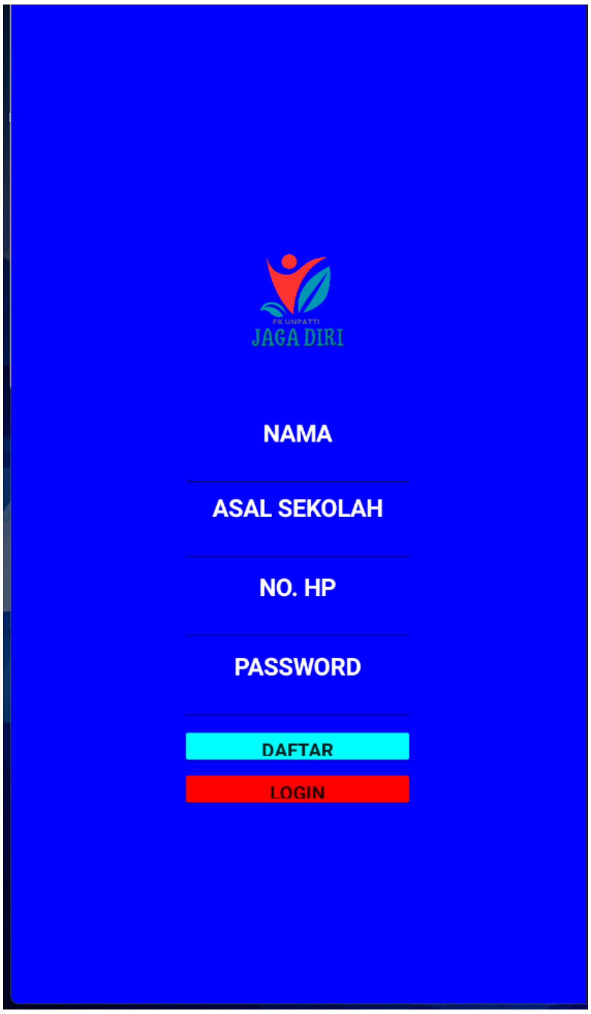

Figure 2. Account List Page Display

Figure 2 shows the account registration form for the “Jaga Diri” application, where users are prompted to enter their personal details, such as name, school, cellphone number, and password, before clicking the 'REGISTER' button to create an account. If the user already has an account, they can simply click the 'LOGIN' button to proceed.

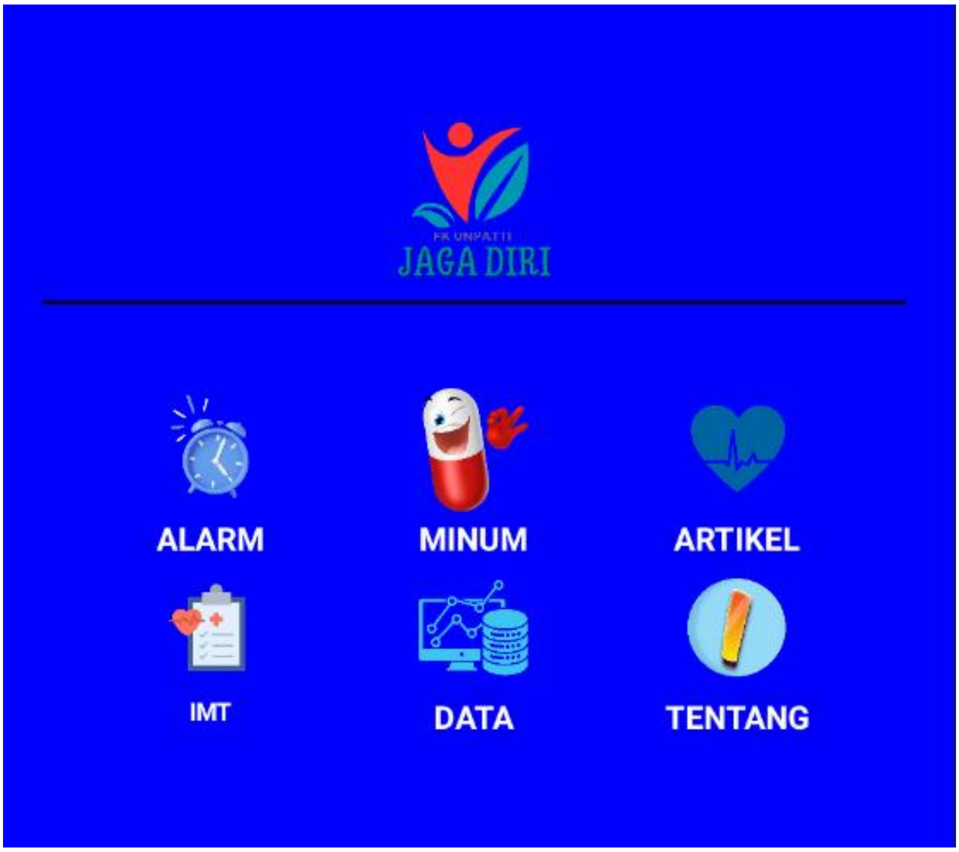

Figure 3. Main Menu page view

The main menu (Figure 3) includes six features available for users to access.

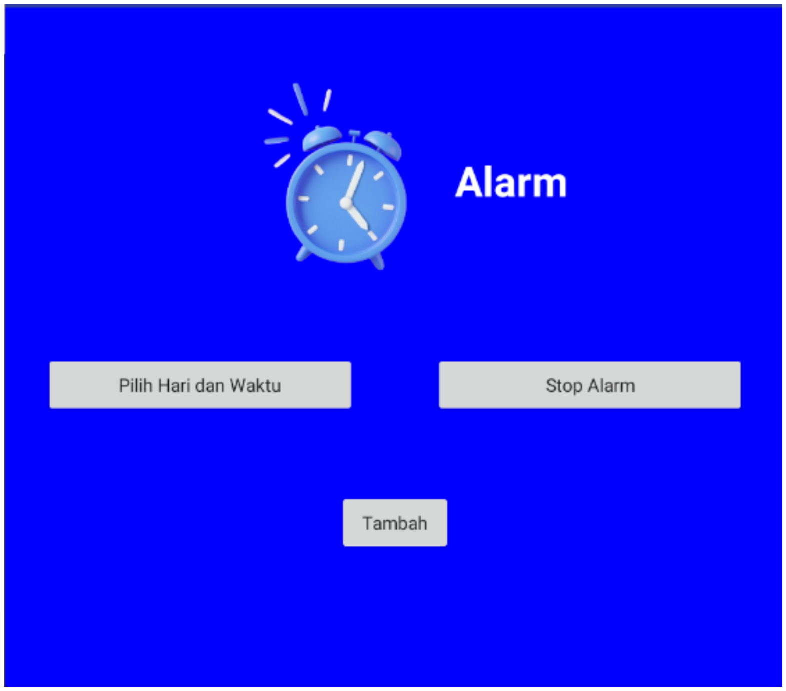

Figure 4. Alarm Feature Display

Figure 4 shows the initial display of the Alarm feature in the “Jaga Diri” application. Users can add alarms by selecting the 'Select Day' and 'Time' buttons, as illustrated in Figures 5 and 6. Once the user selects the desired day and time, the application automatically saves the alarm to the alarm list, as shown in Figure 7. When the time on the user’s smartphone matches the alarm setting in the “Jaga Diri” application, a notification is automatically sent to the user’s smartphone, appearing in the notification bar, as shown in Figure 8.

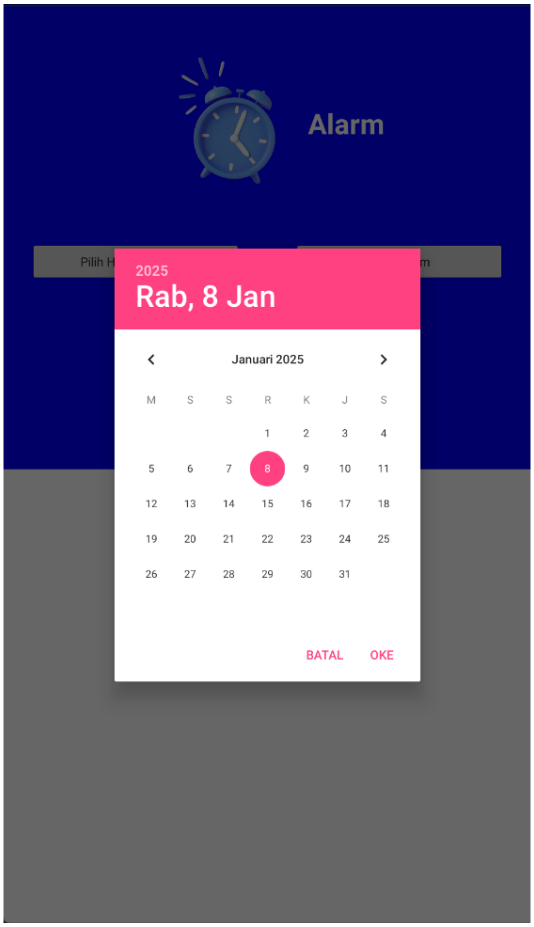

Figure 5. Show "Select day and Time"

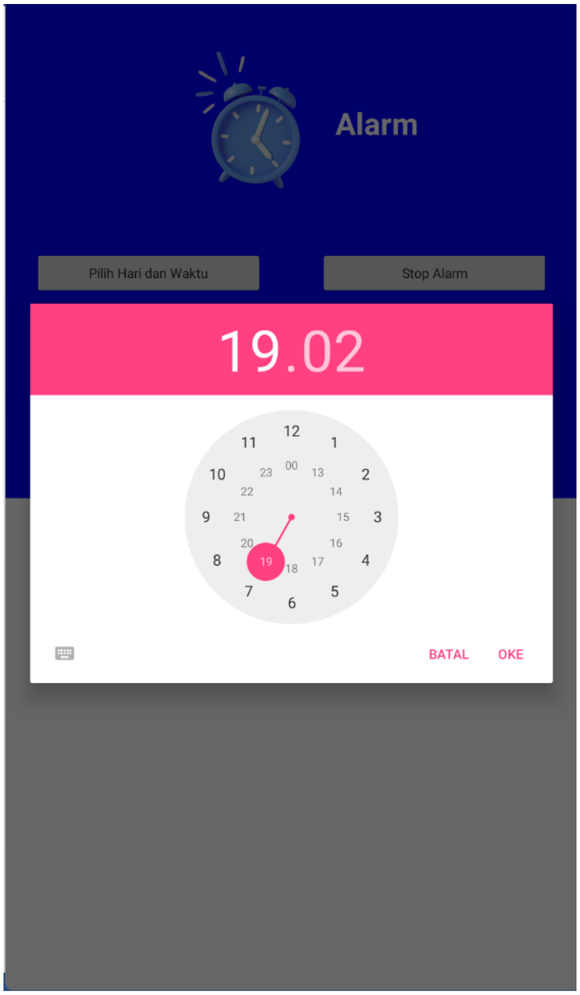

Figure 6. Show "Select day and Time"

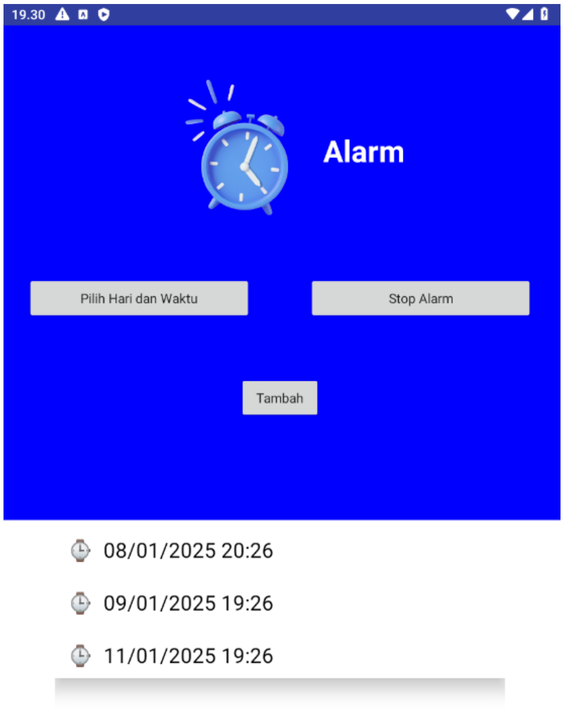

Figure 7. Alarm settings list view

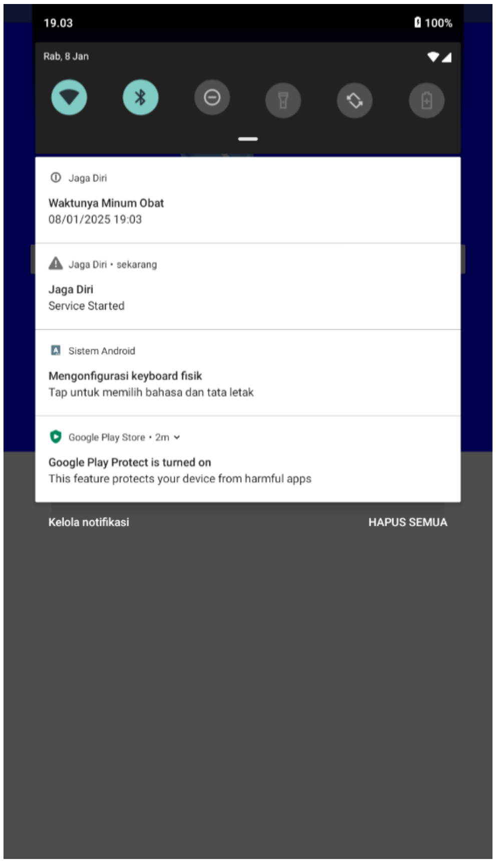

Figure 8. Alarm notification display of the “Jaga Diri” App

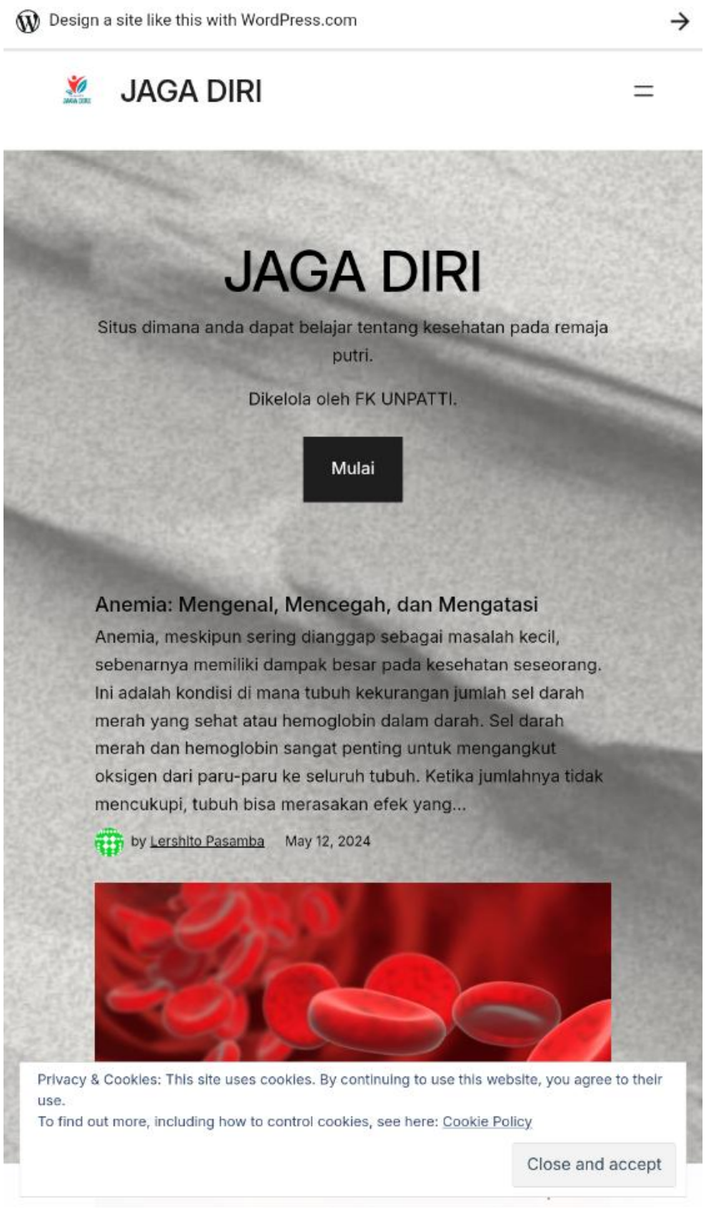

Figure 9. Article Feature Display

Figure 9 is a display of the Article feature which provides information related to Anemia

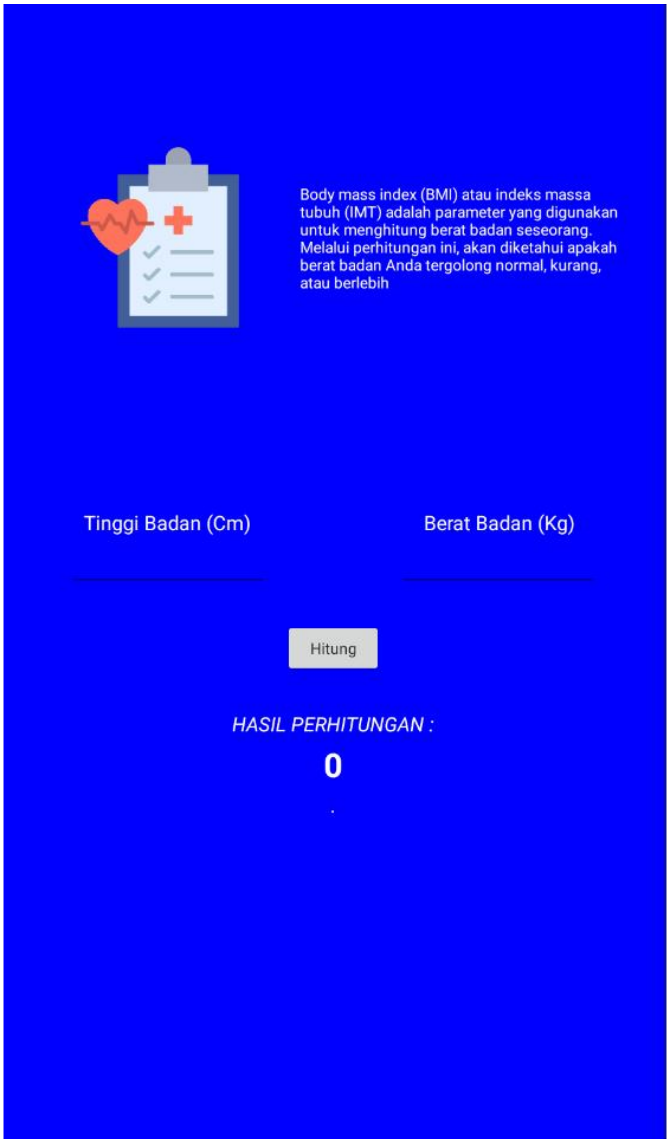

Figure 10. IMT Feature Display

Figure 10 displays the BMI (Body Mass Index) feature of the application, which allows users to calculate their own BMI. This feature includes two input fields that must be filled out by the user: 'Height (Cm)' for the user's height in centimeters and 'Weight (Kg)' for the user's weight in kilograms. After entering the required information, the user can click the 'Calculate' button to view their BMI calculation results, along with a description of their BMI value, as shown in Figure 11.

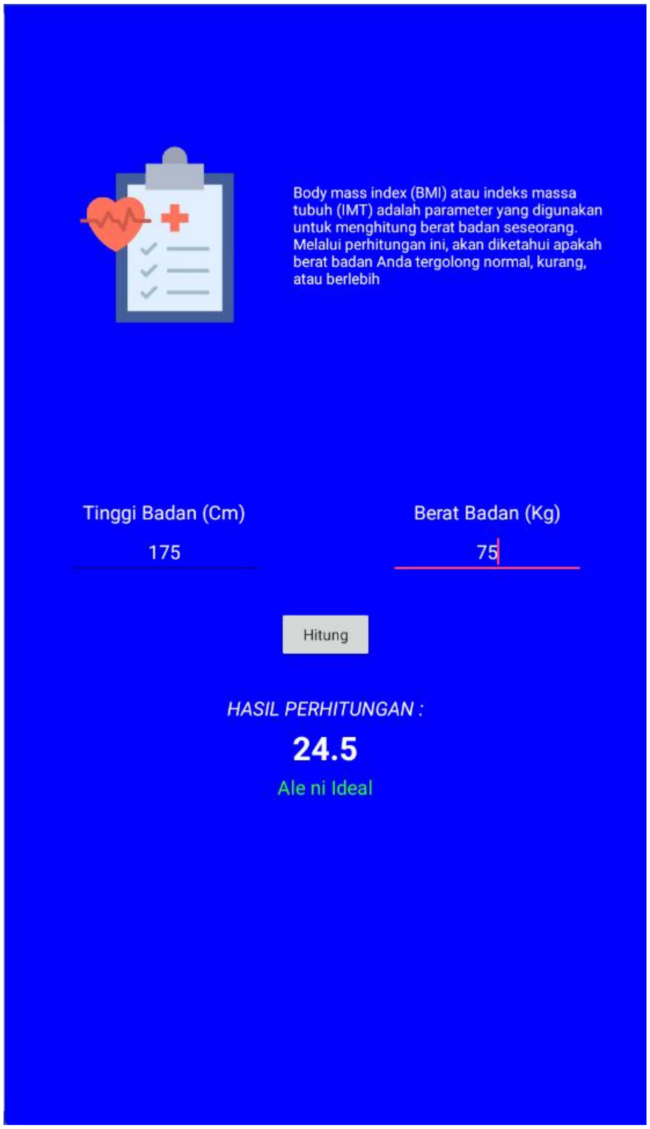

Figure 11. Display of IMT Feature Calculation Results

Figure 11 shows the results of a user's BMI calculation based on a height of 175 cm and a weight of 75 kg, yielding a BMI of 24.5 with the description *'ale ni ideal*,' meaning normal. The BMI descriptions in the 'Jaga Diri' application are presented in the Maluku regional language and are categorized into four levels: *'ale ni kurus*,' meaning thin (BMI<18.5); *'ale ni ideal*,' meaning normal (BMI=18.5–25.0); *'ale ni gemuk*,' meaning overweight (BMI = 25.1–27.0); and *'ale su obesitas*,' meaning obese (BMI>27.0).

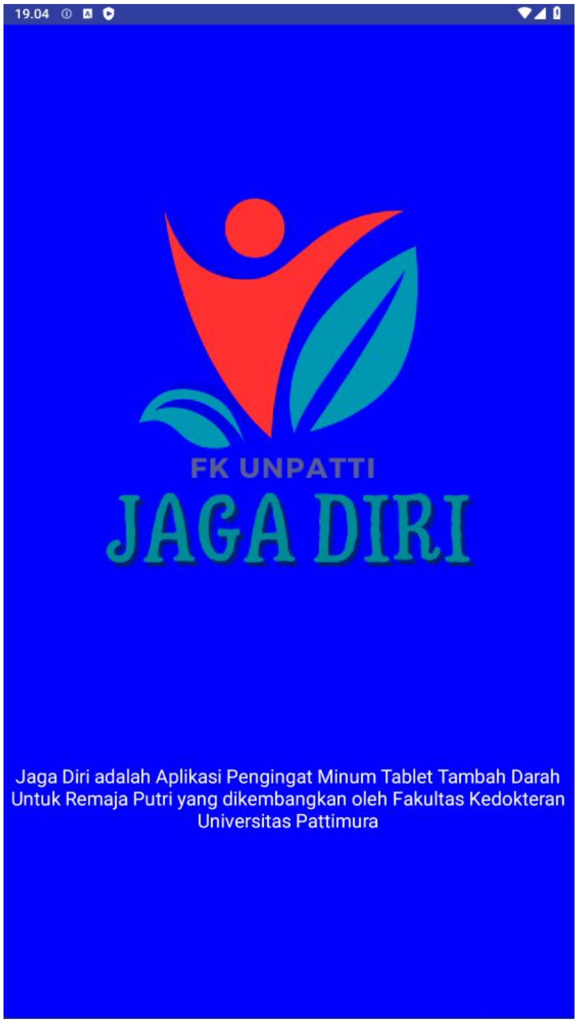

Figure 12: Display of the About Feature

Figure 12 shows the description of the Jaga Diri application.

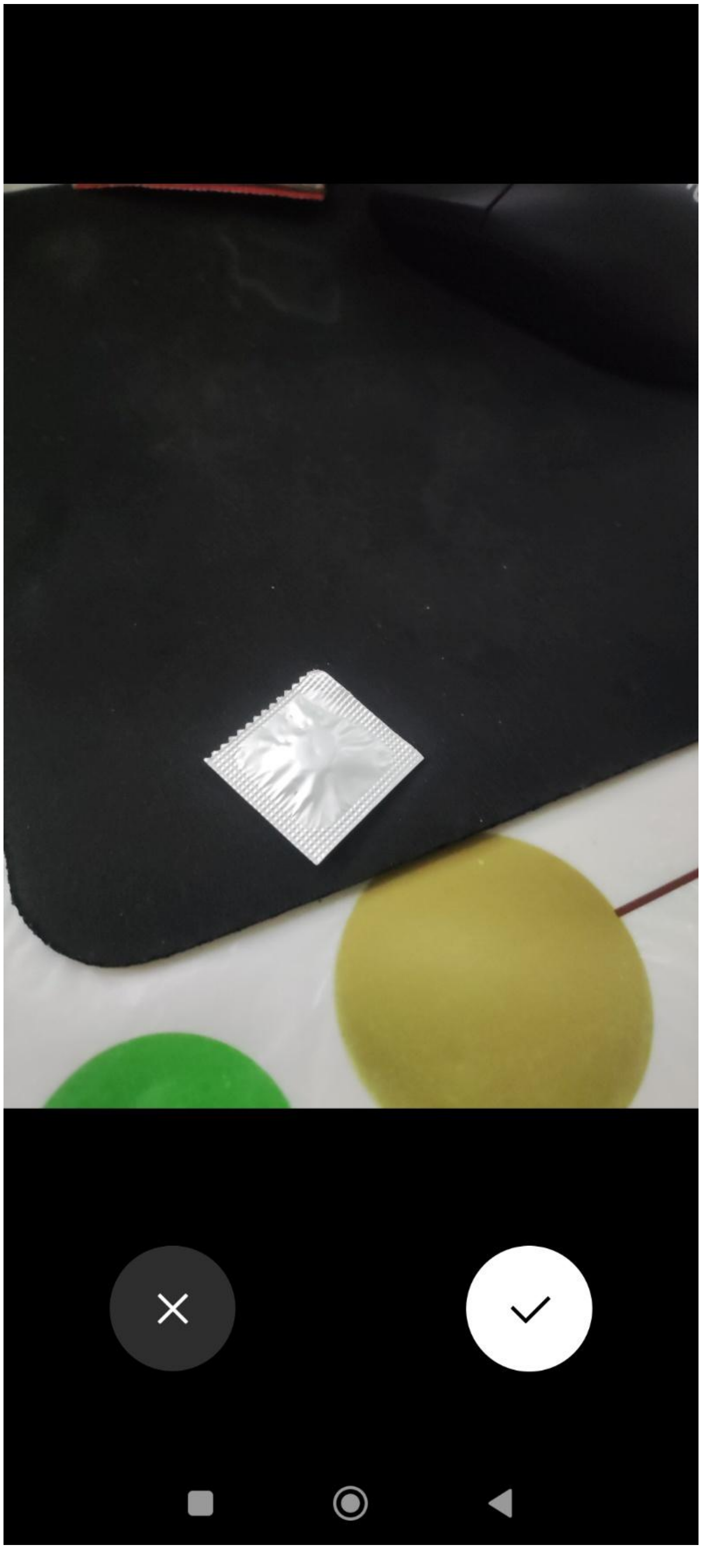

Figure 13. Camera View of Drinking Feature

In the Drinking Feature option on the main menu, the user's smartphone automatically activates the camera to document evidence of the user taking iron-folic acid tablets (TTD), as shown in Figure 13. If the photo evidence captured is unsatisfactory, the user can press the 'cross' button to retake the

photo. Once the photo is deemed appropriate, the user can press the 'check' button, which will automatically save the evidence to the evidence page, as shown in Figure 14.

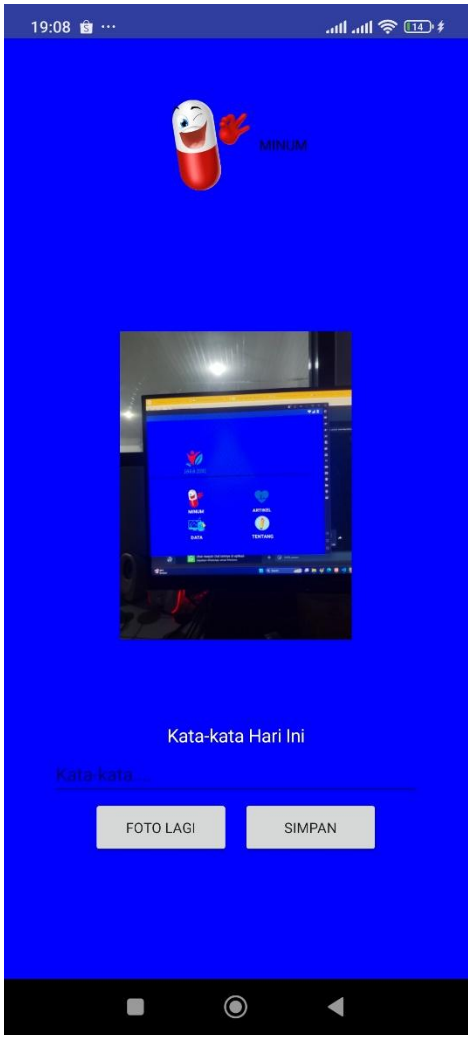

Figure 14. Display of TTD drinking proof page

Figure 14 displays the documentation of the user's proof of taking TTD, with an added feature called 'Today's Words' to enhance the documentation process. On this page, users have the option to retake the photo by selecting the 'PHOTO AGAIN' button or to save the proof of taking TTD by pressing the 'SAVE' button.

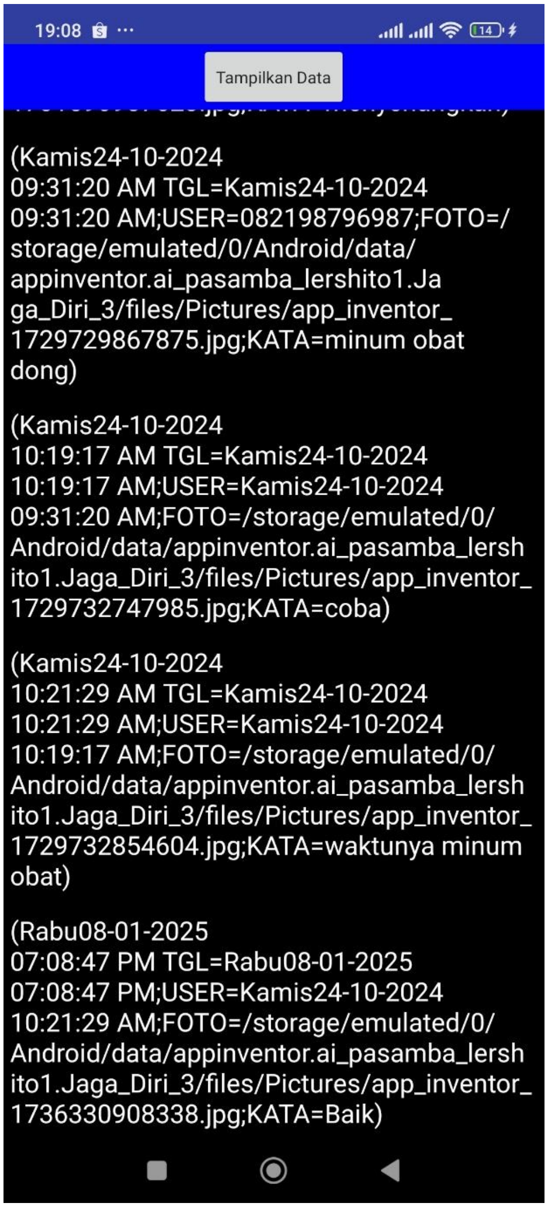

Figure 15: Data Feature Display

Figure 15 shows the results of the user's input for Proof of Drinking TTD. The data stored by the “Jaga Diri” application includes the time and date of medication intake, the directory for storing photo documentation of the proof, and the associated 'Today's Words' saved by the user
